# Biphasic Bioceramic Obtained from Byproducts of Sugar Beet Processing for Use in Bioactive Coatings and Bone Fillings

**DOI:** 10.3390/jfb14100499

**Published:** 2023-10-09

**Authors:** Miguel Suffo-Pino, Miguel Ángel Cauqui-López, Celia Pérez-Muñoz, Daniel Goma-Jiménez, Natalia Fernández-Delgado, Miriam Herrera-Collado

**Affiliations:** 1 Department of Mechanical Engineering and Industrial Design, High Engineering School, Campus Río San Pedro, University of Cádiz, Puerto Real, 11510 Cádiz, Spain; celia.perez@uca.es; 2 Department of Materials Science and Metallurgical Engineering and Inorganic Chemistry IMEYMAT, Campus Río San Pedro, University of Cádiz, Puerto Real, 11510 Cádiz, Spain; miguelangel.cauqui@uca.es (M.Á.C.-L.); dani.gomajimenez@gm.uca.es (D.G.-J.); natalia.fernandezdelgado@uca.es (N.F.-D.); miriam.herrera@uca.es (M.H.-C.)

**Keywords:** biphasic bioceramic, hydroxyapatite, Carbocal^®^, viability, bioactive coating, bone cement

## Abstract

This study focuses on developing hydroxyapatite synthesized from a CaCO_3_-rich byproduct of sugar beet processing called Carbocal^®^ using a hydrothermal reactor. The purpose of this biomaterial is to enhance the osteoinductivity of implantable surfaces and serve as a bone filler, providing a sustainable and economically more affordable alternative. This research involved compositional analysis and micro- and macrostructural physicochemical characterization, complemented with bioactivity and live/dead assays. The biphasic nature of the Carbocal^®^-derived sample was significant within the context of the bioactivity concept previously proposed in the literature. The bioactivity of the biomaterial was demonstrated through a viability test, where the cell growth was nearly equivalent to that of the positive control. For comparison purposes, the same tests were conducted with two additional samples: hydroxyapatite obtained from CaCO_3_ and commercial hydroxyapatite. The resulting product of this process is biocompatible and possesses properties similar to natural hydroxyapatite. Consequently, this biomaterial shows potential as a scaffold in tissue engineering and as an adhesive filler to promote bone regeneration within the context of the circular bioeconomy in the geographical area proposed.

## 1. Introduction

Any foreign body in the organism encourages the development of infections and, consequently, the rejection of the body [1]. This phenomenon is particularly frequent in dental implants. The glycoproteins present in the saliva coating these implants facilitate colonization by microorganisms (especially streptococci), which then proliferate in the oral cavity [2,3]. A similar situation occurs with prostheses in traumatology, for example, in total knee arthroplasty (TKA) or hip arthroplasty (THA), where the materials used have been based on stainless steels, titanium alloys, Cr-Co-Ni alloys, Vitalium^®^, and other metals that are dangerous for humans [4]. In this case, the problem arises from a lack of proven biocompatibility in the medium or long term. In these implantable components, the objective is to enhance their longevity and promote their integration into the bone. This is achieved by incorporating a material that is cemented to the metallic or non-metallic implant surfaces, serving as a true osteoinductive barrier and providing protection against infections. Despite the high success rate of these procedures, there are still 19% of patients who report dissatisfaction with the procedures they have undergone and who have prompted a review [5].

One strategy to be considered is the extraction of biocompatible implantable biomaterials from biowaste, to protect both environmental issues and human wellness. Since the first studies carried out [6,7,8,9,10], it has been proven that nature has provided biomaterials that, through physico-chemical processes, have generated structured biocompatible compounds such as Hydroxyapatite (HA) [11,12].

In the particular case of HA-based coatings, diffusion and plasma spray methods have been applied [13]. HA is a mineral that occurs naturally in bones and teeth, and it has been used as a bone filler in surgical interventions for years. HA has been widely used in biomedical applications due to its excellent biocompatibility, osteoconductivity, and similarity to the mineral component of natural bones [14]. However, the traditional process of obtaining HA involves the use of animal bones, which can lead to ethical concerns and environmental issues [15]. In addition, commercial hydroxyapatite has a very high cost of close to EUR 400/100 g. This, in addition to the fact that approximately 6000 interventions that require the use of bone filler are performed in the region of Andalusia (Southern Spain) each year, has led researchers to explore alternative sources of HA, with a particular focus on circular bioeconomy, sustainability, and clean production.

One promising approach is the use of a waste product from the sugar industry to create HA, but there are few studies on its use [16]. In reference [17], the authors obtained HA through the wet precipitation method of CaCO_3_ refined from sugarcane filter cake (SFC) and orthophosphoric acid. The sugar industry, irrespective of the beet variety [18,19], generates high volumes of byproducts/co-products/waste (bycow hereinafter), amounting to 80% of the original raw material [20]. Approximately 140 g of sugar can be obtained from each ton of beets, with the rest being recoverable byproducts/residues [21]. The bycows that are suitable for recovery as part of other value-added products are the following ones: “Carbocal^®^”, “dried pulp”, and “sugar beet leaves” (vegetable matter) [21]. At present, these bycows have outlets as soil amendment and animal feed and also in the production of cement for the construction sector, but a large fraction of them tend to end up in landfill sites.

Carbocal^®^, being a commercial byproduct from beet sugar processing, is similar to filter cake [16]. It contains more than 80% CaCO_3_ (greater than the aforementioned references), apart from some trace elements, and has a low market price and that makes it more attractive for use in biomedical applications. This waste can be transformed by a reaction to create synthetic HA.

Using sugar industry waste-derived HA as a bone filler in surgical interventions and for covering prostheses can enhance the success rates of these procedures while reducing the potential complications. Furthermore, the use of a waste-derived HA contributes to sustainability efforts. It helps reduce the demand for natural resources and minimizes the need for the environmentally harmful extraction processes typically associated with traditional HA production.

By utilizing a waste product from the sugar industry, HA production can contribute to waste reduction and promote the principles of circular economy, thus moving towards the fulfilment of the Sustainable Development Goals required by the European Union. Instead of being sent to a landfill site, it is repurposed into a valuable material, reducing the environmental impact associated with waste disposal. By implementing eco-friendly extraction techniques, minimizing the use of chemicals, and optimizing energy consumption, the overall environmental footprint of the production process can be reduced.

In this view, the present work develops a hydroxyapatite-based biomaterial aimed at use as a bone filling material and priming implantable surfaces to make them osteoinductive. The biomaterial is synthesized from a CaCO_3_-rich byproduct of sugar beet processing called Carbocal^®^ using a hydrothermal reactor.

## 2. Materials and Methods

### 2.1. Materials

For this study, Carbocal^®^ was acquired from the company AB Azucarera, a sugar beet factory in the province of Cádiz (Andalusia, Spain) in 2022. The volume of annual production and its consideration as waste, with no guaranteed valorization, are factors that make Carbocal^®^ a suitable byproduct. Table 1 summarizes its chemical composition and generation process.

Two different sources of CaCO_3_ were used: Carbocal^®^ and commercial CaCO_3_ (supplied by Sigma-Aldrich (now Merck, Darmstadt, Germany)). It is a suitable bycow due to its nature, volume of annual production, and consideration as a waste product with no guaranteed valorization. Reagent-grade sodium dihydrogen phosphate monohydrate provided by Sigma-Aldrich (now Merck, Darmstadt, Germany) was used as a source for phosphate.

### 2.2. Methodology

Hydroxyapatite samples were prepared using hydrothermal phosphatization of CaCO_3_, according to the following reaction [7,19]:

10 CaCO_3_ + 6 NaH_2_PO_4_ + 2 H_2_O → Ca_10_(PO_4_)_6_(OH)_2_ + 6 NaHCO_3_ + 4 H_2_CO_3_ (1)

Briefly, the desired amounts of Carbocal^®^ (or CaCO_3_) and NaH_2_PO_4_ (nominal Ca/P ratio of 1.67) were added to 100 mL of milliQ water under continuous stirring and kept for 2 h. The dissolution of the precursors was carried out in a 200 mL glass beaker at 25 °C using a magnetic stirrer (Thermo Fisher-Scientific, Waltham, MA, USA) with a stirring speed of 700 rpm, with a Teflon-coated magnetic bar (Labbox, Barcelona, Spain) with a length of 40 mm and a width of 8 mm. This solution was then transferred to a 250 mL Teflon vessel using different portions of water, reaching a total volume of 200 mL. After total dissolution of both reactants, the pH reached was 7. The Teflon vessel was hermetically sealed, placed in a stainless-steel hydrothermal reactor (Mecaprec, Cádiz, Spain), and heated in an oven (J.P. Selecta, Barcelona, Spain) at 200 °C for 24 h. After cooling to room temperature, the solids were collected by centrifugation (3 min) and washed with milliQ water (Wasserlab, Navarra, Spain). A ThermoFisher (Thermofisher, Waltham, MA, USA) Sorvall ST16 series centrifuge with a swinging Bucket Rotor TX-400 model was used. This rotor can load four vessels up to 570 g and has rotation speeds of up to 5000 rpm. The washing and centrifugation process was repeated three times. Finally, the solids were oven-dried at 110 °C for 24 h, crushed in an agate mortar, and sieved using a 75-micron mesh.

Samples obtained from Carbocal^®^ and CaCO_3_ are hereinafter referred to as HAcc and HAct, respectively. For comparison, a commercial hydroxyapatite supplied by Aldrich was also studied (HAref).

- Microstructural Properties, Chemical Characterization, Structural Properties, and Thermogravimetric Analysis

Fourier Transform Infrared Spectroscopy (FTIR) provided information related to the presence or absence of specific functional groups, as well as the chemical structure of the present compounds. A Bruker^®^ Vertex 70 instrument equipped with a DTGS detector (Bruker, MA, USA) was used. It allowed for the recording of FTIR spectra of solids in the mid- and near-infrared ranges. For the acquisition of spectra, a standard spectral resolution of 4 cm^−1^ within the range of 4000–400 cm^−1^ was used, as well as 64 accumulations per sample.

The materials were analyzed, applying scanning electron microscopy (SEM) techniques to investigate the morphology and the distribution of the phases forming the structures. Images were acquired using a Nova NanoSEM microscope from FEI Company, equipped with an X-ray detector (Thermo Fisher Scientific, Hillsboro, OR, USA) working at 20 kV and at a current of 68 µm. In order to protect the samples from the electron beam, the material of interest was coated with 15 nm of Au by a 208 HR Sputter Coater Cressintong system (Leica, Wetzlar, Germany) [23].

The chemical compositions of the different HA samples were determined by inductively coupled plasma atomic emission spectroscopy (ICP-AES) using a Thermo Elemental plasma atomic emission spectrometer (Intrepid model, Thermo Scientific, Waltham, MA, USA).

Powder XRD patterns were recorded on a Bruker D8 Advance A-25 diffractometer (Bruker Corporation, Billerica, MA, USA), working in Bragg–Brentano geometry using Cu-Kα as the radiation source. X-ray diffractograms were collected at room temperature over the 2-theta range from 15 to 60°, with a stepwise increment of 0.03° and an acquisition time of 3 s per step. The XRD patterns were evaluated for solid-phase distribution and crystallite sizes using Profex-5 software (version 5.2) at a refinement confidence for statistical evaluation of ꭓ^2^ ≤ 1.5 [24].

TGA was carried out to investigate the thermal stability of the HA samples. These experiments were performed in a thermogravimetric analyzer (TAinstruments Q50) (TA instruments, New Castle, DE, USA). Fresh samples were introduced into a microbalance pan and heated up to 900 °C with a temperature ramp of 10 °C/min under an air/nitrogen mixture (60/40 *v*/*v*, with a total flow of 100 mL/min).

B. Density, Relative Density, and Textural Properties

The density (n = 3) was determined according to method B of standard ASTM D 854-2006 [25]. A pycnometer (250 mL) and distilled water were used. Mass measurements were carried out on a 0.001 g-precision balance, and the volume was calculated from their dimensional values, which had previously been measured with a Vernier caliper. The samples were dried to constant mass in an oven at a constant temperature of 110 °C for 24 h [26,27]. The relative density of the samples was evaluated from the volumetric mass density of pellets, taking the theoretical density of HA as 3.156 g/cm^3^. The volumetric mass density of pellets was estimated using the gravimetric method. The pycnometric density of pellets was measured with a helium pycnometer Ultrapyc 1200 e (Quantachrome Instruments, Boynton Beach, FL, USA) at 20.2 °C. Gaseous helium with a volume fraction of at least 99.9999% was used.

The textural properties of the samples were determined by means of N_2_ adsorption–desorption isotherms using a Quantachrome Autosorb IQ3 (Anton Paar, Graz, Austria). Prior to the measurement, the samples were degassed under vacuum for 6 h at 150 °C to remove physically adsorbed components and other adsorbed gases from the sample surface. The specific surface area (SBET) was calculated using the multipoint BET (Brunauer–Emmett–Teller) method. The pore volume was obtained using the BJH method (Barrett–Joyner–Halenda) using the desorption branch isotherm obtained in the adsorption experiment.

C. Cell Viability Assay

Finally, cell cultures were performed with human fetal osteoblastic cell lines (hFOBs 1.19) in the presence of the HA samples to determine whether the materials affected cell viability. For this cell viability study, hFOBs were cultured in osteogenic media and viability assays were performed at 24, 48, 72 h, and 7 days. In these assays, the cells were incubated with MTS and the absorbance was subsequently quantified. As a negative control, before the process, hFOB cultures were incubated with 70% methanol for 30 min. As a positive control, hFOB cultures without any treatment were used.

The biocompatibility of HA samples was tested with human osteoblastic primary cell hFOB cultures. Normal human fetal osteoblasts were commercial from ATTC (hFOB 1.19 cell line, CRL-11372^TM^) (Manassas, VA, USA).

hFOB cultures were grown in osteogenic media containing Dulbecco’s Modified Eagle Medium culture (DMEM), with 10% fetal bovine serum (FBS) and antibiotic (G480 30 mg/μL). Cells were expanded by incubation at 35 °C in 75 cm^2^ flasks with 5% CO_2_.

Viability assays were performed over 7 days. As a negative control, prior to the labeling process, the hFOB cultures were incubated with 70% methanol for 30 min. As a positive control, hFOB cultures were used without any treatment.

Prior to testing, the materials were sterilized using an autoclave at 120 °C for 20 min. To assess viability, conditioned medium was used, consisting of basal culture medium (DMEM—Gibco) supplemented with 1% FBS (Corning) and 1% antibiotic (G480 30 mg/μL—PAN Biotech) to minimize cell division. Different dilutions of HA samples were formed in this medium to obtain different concentrations, ranging from 15 mg/mL to 100 mg/mL.

For the viability assay, cells were cultured with complete medium in 96-well plates at a concentration of 2 × 10^4^ cells/well. These plates were incubated at 35 °C with 5% CO_2_ for 24 h. After this time, the cells were adhered to the well surface, and the complete medium was removed and replaced with conditioned medium in the cases of positive control (PC) and negative control (NC) and with the HA samples’ solutions in conditioned medium at different concentrations in the rest of the wells, all in duplicate. The plates were incubated again at 34 °C with 5% CO_2_.

To measure viability, the MTS assay was used. Prior to labeling, as a negative control, the cells were incubated with 70% methanol for 30 min. A total of 20 μL of MTS was added to all wells and incubated at 34 °C for 1 h. After this time, absorbance was measured at 490 nm using the Varioskan LUX plate reader (Thermo Fisher Scientific). This process was performed at 24, 48, 72 h, and 7 days of incubation with the different HA solutions. The test was carried out on n = 3 replicas on 3 different samples for each HA solution.

#### Statistical Analysis

Differences in viability between the different HA samples were analyzed comparing the means of the groups with the ANOVA parametric test of comparison of means. To detect differences, a significance level of 0.05 was used. To carry out all the analyses, the SPSS statistical program (IBM Corp, Armonk, NY, USA, 2015, version 24, licensed by the University of Cádiz) was used.

## 3. Results and Discussion

The chemical composition of the materials studied at macroscopical level, mainly regarding the Ca/P ratio, was analyzed using ICP-AES. The results indicated atomic Ca/P ratios of 1.45, 1.66, and 1.59 for the HAcc, HAct, and HAref samples, respectively. These values were lower than the stoichiometric value expected for pure HA (1.67). This effect was particularly significant in the case of the HAcc sample and can be explained in terms of a partial substitution of the PO_4_^3−^ groups by CO_3_^2−^ ions [28], or it may be due to the coexistence of HA with additional phases having different Ca/P ratios (e.g., Ca_3_(PO_4_)_2_, tricalcium phosphate, TCP) [29].

**Figure 1:**
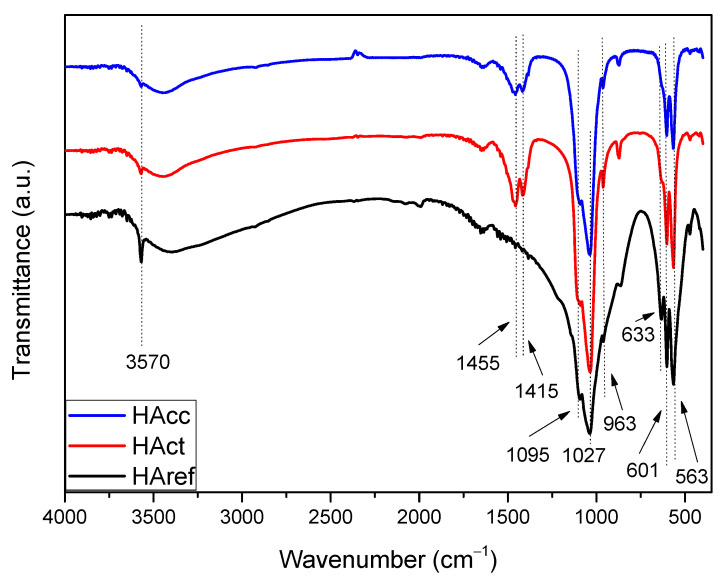
FTIR spectrum corresponding to the HAcc, HAct, and HAref samples.

The infrared spectroscopy technique is commonly used for the characterization of apatite-type materials. Figure 1 shows the FTIR spectrum obtained for the HAcc, HAct, and HAref samples, where all the vibrations characteristic of the HA phase can be identified [30]. Thus, for example, the typical vibrations of the PO_4_^3−^ ions were observed at 963 cm^−^^1^ (ν1), 1027, 1095 (ν3), and 563, 601 (ν4) [30,31,32]. The peaks at 633 and 3570 cm^−^^1^ were assigned to characteristic OH bands of HA [33]. In addition, the peak at 873 cm^−^^1^ and the broad doublet around 1415–1455 cm^−^^1^ indicated the presence of CO_3_^2−^ groups and, in particular, of the so-called B-type groups, which resulted from a partial substitution of PO_4_^3−^ by CO_3_^2−^ ions [34]. This doublet was not visible in the case of the reference sample, suggesting the absence of this type of carbonate in its structure. As is widely known, the HA that forms part of the bone is also a B-type carbonated phase so, in principle, it would be reasonable to expect a positive contribution from this composition in terms of biocompatibility [35]. No additional peaks that may have resulted from the extraneous substitution of functional groups or contamination were detected.

**Figure 2:**
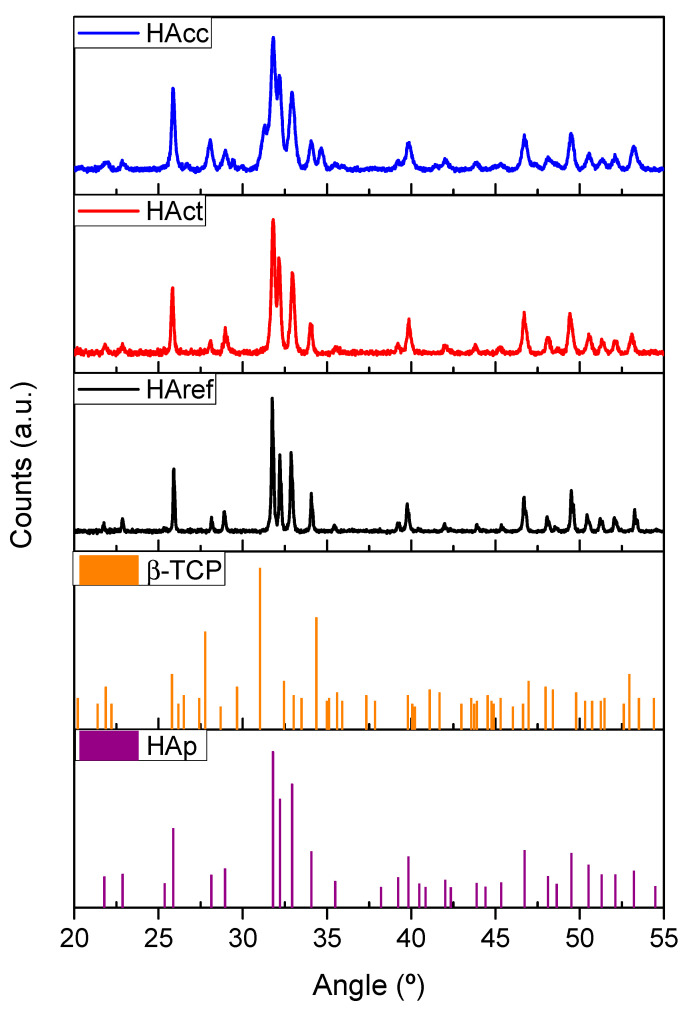
XRD diffractograms from HAcc, HAct, and HAref samples. Standard patterns for pure Hydroxyapatite (HAp) and β-TCP are also included.

The crystalline structures of the investigated materials were unraveled using the XRD technique. Figure 2 shows the XRD diffractograms corresponding to the HAcc, HAct, and HAref samples. The diffractograms revealed the presence of the hexagonal hydroxyapatite structure (HA). This is a unique phase observed for HAct and HAref. However, in the case of the HAcc sample, which was the one prepared using Carbocal^®^, peaks corresponding to β-tricalcium phosphate (TCP) were clearly observed. No additional phases (such as free calcium oxide) were identified.

The results from the XRD analysis, as determined through phase refinement using Profex 5 software (version 5.2), are gathered in Table 2.

These analyses confirmed the biphasic character of the HAcc sample, which showed the following composition: 75% *w*/*w* HA and 24.83% *w*/*w* β-TCP. The lattice parameters estimated for the three samples were typical for hexagonal HA- and β-TCP-type structures [32,36]. Nanometric crystallite sizes ranging to the order of 22–58 nm, similar to the ones reported for HA in bones [37], were estimated for the synthesized samples (HAcc and HAct). The crystallinity was higher in the case of the sample used as a reference (HAref), as deduced from the average crystallite size obtained for this sample.

The biphasic character of the sample prepared from Carbocal residue (HAcc) became important in the framework of the concept of bioactivity proposed by Daculsi in France, and Lynch, Nery, and LeGero in the USA in the 1980s [38]. As is well known, this concept was based on the singular properties of biphasic calcium phosphate ceramics (so-called BCP). BCP bioceramics consist of mixtures of hydroxyapatite (HA) (more stable) and beta-tricalcium phosphate (β-TCP) (more soluble) of varying HA/b-TCP ratios. This material, which is structurally similar to the HAcc prepared in this work, gradually dissolves in the body, generating new bone formation as it releases Ca^2+^ and PO_4_^3−^ ions into the biological environment, which promotes the mineralization and formation of new bone. In addition, both phases are expected to be biocompatible, which is an essential property to avoid adverse reactions and promote integration with the surrounding tissue. BCP is commercially available in Europe, Brazil, Japan, the USA, and Australia as a bone graft or as bone substitute materials for orthopedic and dental applications under various trademarks (BCP^®^, MBCP^®^, Triosite^®^, Hatric^®^, Eurocer^®^, Biceram^®^, Bicalfoss^®^).

**Figure 3:**
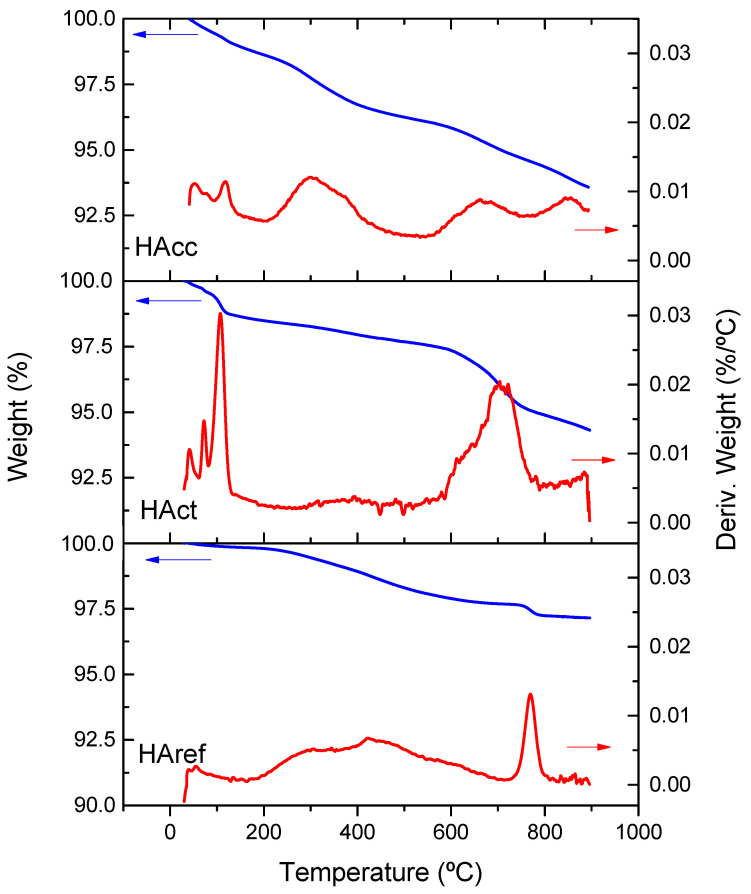
Thermogravimetric curve of the HAcc, HAct, and HAref samples.

Figure 3 shows the thermogravimetric curves obtained for the three samples heated from room temperature up to 900 °C. The weight losses were about 5–6% for the synthetized samples (HAcc and HAct), while for the reference it was about half (2.5%). For better identification of the different weight loss processes, the derivative curves were also included. The small peaks appearing in the 35–200 °C range corresponded to adsorbed water, which was almost negligible in the case of the reference sample. The release of water from the lattice was associated with the weight loss process occurring in the 200–400 °C range. As can be seen, this process was much faster in the case of the hydroxyapatite prepared from Carbocal^®^. Two additional peaks at higher temperatures (600–900 °C) were probably related to the dihydroxylation and decarboxylation processes [39].

**Figure 4:**
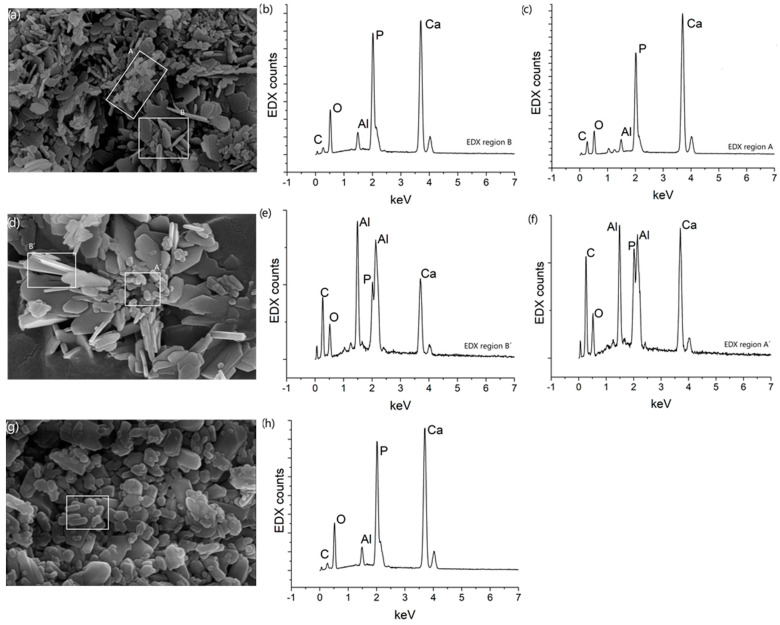
SEM micrographs of HAcc (**a**), HAref (**d**), and HAct (**g**) samples. EDX spectra taken from the regions marked in the SEM micrographs, with (**b**) and (**c**) corresponding to HAcc sample, (**e**) and (**f**) corresponding to HAref sample, and (**h**) corresponding to HAct sample.

Figure 4 shows SEM micrographs of the materials analyzed in the present work. Figure 4a corresponds to the HAcc sample and shows that two main morphologies were present in the HAcc powder, including flat sheets (region marked as B) and more spherical morphologies of smaller sizes (region marked as A). In comparison, the reference sample, HAref, also showed the two morphologies mentioned (see Figure 4d), although it seemed that the flat sheets were of larger size. Regarding the HAct sample, SEM imaging showed a wider distribution of morphologies and sizes, as exhibited in Figure 4g. In the literature, flake-like morphologies have been observed upon calcination of waste poultry eggshells, and it has been demonstrated that it is not HA but another type of calcium phosphate-based biomaterial [40]. In that study, micrographs of powder obtained at a higher temperature and identified as a mixture of HA and monetite using XRD showed a more rounded morphology, similar to that observed in Figure 4a,d. EDX analyses were carried out to investigate the composition of the studied materials. These demonstrated that all the samples consisted of Ca, P, and O, as expected (it should be mentioned that the SEM holder was composed of an alloy of Al and Mg, and because of that, these elements also appeared in the spectra in different amounts based on the proximity of the holder to the region of analysis). The EDX analyses that were carried out also showed that the composition of the materials varied for the different morphologies found. Figure 4b,e correspond to the flat sheets in the HAcc and HAref samples, respectively, and here it was observed that the P/Ca ratio was larger than that found in Figure 4c,f, corresponding to the spherical morphologies in the mentioned samples. In the HAct sample, because the different morphologies were mixed in the powder, separate spectra for the different morphologies were not possible to acquire (see Figure 4h). The family of phosphate compounds is quite large, having different Ca/P ratios according to the material composition. In particular, the Ca/P ratio for HA was 1.67. Different Ca/P ratios in the range of approx. 1.5–2 were found in our EDX-SEM analyses of the different materials, with a ratio of 1.6 being found for the round morphologies in a few cases, which corroborates the result of 1.56 obtained using XRF compositional analysis and the presence of Mg at 1.3% [26]. Care should be taken in the quantification of the composition of mixed compounds using SEM, as signals from adjacent materials can be obtained together with that related to the compound of interest, leading to an averaging of the element content. Our results point to the existence of HA mixed with other phosphates, which is somewhat expected. In the literature, compounds with different compositions are obtained when extracting HA from waste materials. For example, HA synthesized from non-separated biowastes (animal bones) using heat treatments showed Ca/P ratios in the range of 1.58–1.94% for sintered samples, with the ratio for the raw materials being 2.79 [41]. In a different study, the ratios of Ca/P were found to be 1.56 and 1.88 for treated pig bone and as-synthesized HA, respectively, at 1000 °C [41]. In a study focused on the synthesis of HA from marine shell waste, Ca/P ratios of between 2.1 and 1.75 were obtained, and the difference from the theoretical ratio of 1.67 was attributed to traces of carbonate still present in the samples [42]. In other cases, however, HA has been obtained with a lesser amount of other compounds, such as when is it obtained from waste bovine bone using an alkaline digestion method, which provided a Ca/P molar ratio of 1.75, which is similar to previous reports using fresh bovine bones [43]. Regarding a potential compatibility of the material obtained with human tissue, it should be highlighted that no heavy metals were found in the EDX analyses performed, which is promising for biological applications.

### 3.1. Density, Relative Density, and Textural Properties

According to the pycnometer test, taking into account the different ratios determined with EDX and ICP, the value obtained for the average density of the grains was 2.69 g cm^−3^ ± 0.005, which is quite close to the mineral content of a healthy trabecular bone [2,44,45,46]. The relative density was 85.45%, which is similar to [3,32] for 100% HA before sintering and slightly higher than that obtained in [4,35], although in those studies, composites with a lower percentage of HA by weight were used. Considering that the synthesized HA came from Carbocal^®^ and was basically composed of CaCO_3_, whose density is averaged at 2.71 g cm^−3^ (density of calcite) [5,47], and CaHPO_4_ 2H_2_O = 2.31 g × cm^−3^ [48], a maintenance of the initial density of the composite after undergoing the reaction and drying processes was observed.

**Figure 5:**
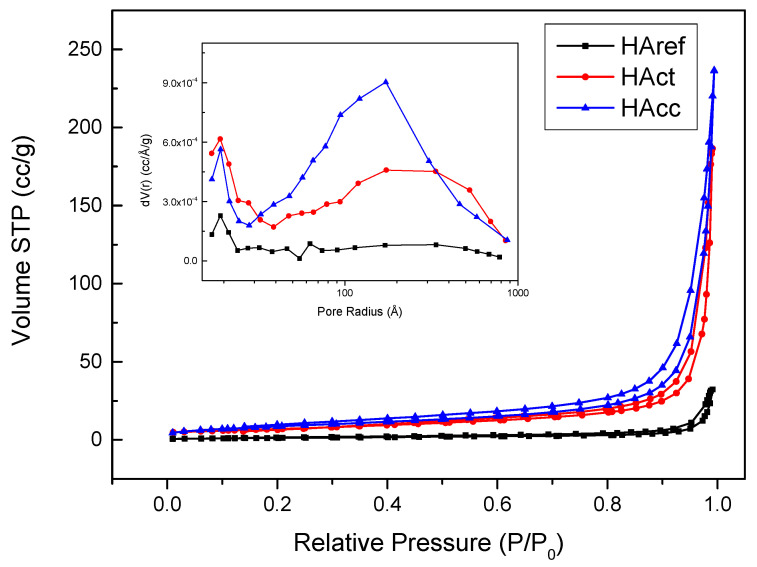
Adsorption—desorption isotherms obtained for the HAcc, HAct, and HAref samples and distributions of pore radius.

The textural properties of HAcc can play an important role in the integration of these materials into nanocomposite matrices for different purposes, such as for the fabrication of bone prostheses. For the textural characterization of the samples, the technique of volumetric adsorption–desorption of nitrogen at a temperature of 77K was used. The adsorption–desorption isotherms are shown in Figure 5. As can be seen, the adsorption branch resembled a Type IV isotherm according to the IUPAC classification [49]. Moreover, an H3-type hysteresis loop was observed. Loops of this type are typical of non-rigid aggregates of plate-like particles [50]. The calculated BET surface areas were 32, 27, and 5 m^2^ g^−1^ for the HAcc, HAct, and HAref samples, respectively. The lowest value obtained for HAref agreed with its higher crystallinity obtained using XRD. Values between 5 and 44 m^2^ × g^−1^ (depending on the reaction time) were reported for a hydroxyapatite synthesized using CaCO_3_ and NaH_2_PO_4_ as precursors [51].

The distributions of the pore radii were plotted according to the BJH nitrogen desorption (inset in Figure 5). Peaks centered over 20 Å and 200 Å were observed, the latter being of higher intensity, mainly in the case of the HAcc material. As shown in the figure, the porosity of the reference sample was rather low, which was in good agreement with its very low surface area.

### 3.2. Cell Viability Assay

**Figure 6:**
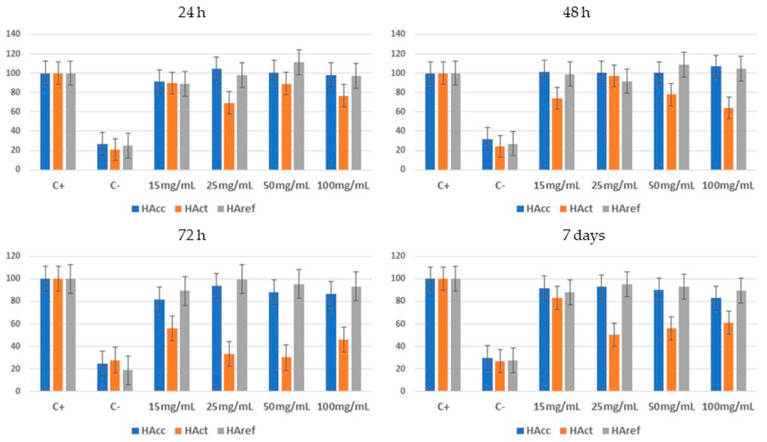
Human osteoblast viability after 24 h, 48 h, 72 h, and 7-day incubation with different concentrations of HA samples. Culture medium without HA was used as the positive control (C+ 100% viability), while 70% methanol was used as the negative control (C−).

None of the HA samples or concentrations used significantly affected the viability of the cell line tested according to the results obtained from the MTS assay (Figure 6). Taking the positive control as 100% viability, no significant decrease in cell viability was found at any of the concentrations tested. On the contrary, the negative control showed an absorbance that did not exceed 40% in any of the cases.

As can be seen in Figure 6, osteoblast culture in the presence of HAct, which was obtained from CaCO_3_, resulted in a lower viability in all cases. The ANOVA test showed that there were significant differences between samples (F = 28.175, p < 0.05), while a post hoc Tukey test found a significant reduction in cell viability in the presence of HAct. However, in the presence of HAcc, which was obtained from Carbocal^®^, the viability was similar to the viability in the presence of commercial HAref at all times measured. This similar viability result may be due to the available surface area of the HAcc, which was obtained from Carbocal^®^.

It is worth mentioning that the presence of HAcc, at certain concentrations, promoted cell growth, with values that exceeded the positive control without these differences being significant.

**Figure 7:**
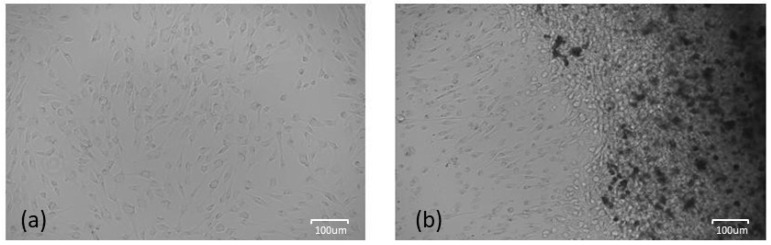
(**a**) Osteoblast cell culture positive control. (**b**) Osteoblast cell culture in the presence of HAcc.

On the other hand, the presence of HAcc in the osteoblast culture did not produce a halo of cell growth, as shown in Figure 7. While in the positive control, the cells were homogeneously distributed throughout the well, in the presence of HAcc, the cells tended to cluster around the HAcc. Thus, there was no halo of cell growth around the material, and cells were found all over the well. This is beneficial, as metallic prostheses can lead to aseptic loosening of the prosthesis due to the lack of bone cell growth around it [52].

## 4. Conclusions

Orthopedic surgery and traumatology (OST) continues to face challenges related to the biocompatibility of the materials used, with 19% of patients expressing dissatisfaction with the procedures performed. The present work describes the production process of HAcc from a byproduct of sugar beet processing called Carbocal^®^. HAcc has been proven to present similar physicochemical and microstructural properties to those of natural HA. This ensures that it provides sufficient support and stability when used as a bone filler or as a prosthesis cover, mimicking the characteristics of natural bones. This shows a positive contribution from this composition in terms of biocompatibility due to the presence of a B-type carbonated phase. It has demonstrated the same cell viability as natural HA when compared to a commercial HA. This means that it supports the growth and proliferation of osteoblasts, the cells responsible for bone formation. The biocompatibility of this material ensures that it integrates well with the surrounding tissues, minimizing the risk of adverse reactions and promoting successful bone regeneration.

Since the sugar industry generates around 200,000 tons per year of Carbocal^®^, the use of synthetic HA derived from this waste product offers a sustainable and environmentally friendly alternative to traditional HA for bone filler and prosthesis covering applications. Its ability to promote the growth of osteoblasts, combined with its favorable material properties, makes it an attractive alternative with a reduced environmental impact compared to traditional methods of HA production, contributing to the circular bioeconomy by utilizing waste materials in a resource-efficient manner.

## 5. Patents

A patent registration application related to this manuscript will be filed.

## Figures and Tables

**Table 1:** Characteristics of Carbocal^®^ [22].

Morphology	Powder
Genesis	Purification process of juice sweetened with lime hydroxide and CO_2_
Chemical composition	>80% CaCO_3_; 7% organic matter; oligo-elements (N, K_2_O, P_2_O_5_ and Mg); assimilable organic acids
Humidity	<35%
Production	20,000 average annual tons

**Table 2:** Mineral phase distribution (% *w*/*w*), network parameters, and crystallite sizes as determined by phase refinement analysis of XRD diffractograms.

Sample	Hydroxyapatite (HA)			β-Tricalcium Phosphate (TCP)		
	% *w*/*w*	Lattice Parameters (Å)	Crystallite Sizes (nm)	% *w*/*w*	Lattice Parameters (Å)	Crystallite Sizes (nm)
HA_cc_	75.17	a: 9.4160	(1,0,0):22.2	24.83	a:10.3523 c: 37.1275	(1,1,1):42.4
		c: 6.8877	(0,0,1):39.5			
HA_ct_	100	a: 9.4112	(1,0,0):39.8	0	--	--
		c: 6.8982	(0,0,1):58.2			
HA_ref_	100	a: 9.4288	(1,0,0):85.9	0	--	--
		c: 6.8728	(0,0,1):150.8			

## Data Availability

The authors declare that the data from the current study will be made available on request.
